# Postpartum haemorrhage: Case definition and guidelines for data collection, analysis, and presentation of immunization safety data^☆^

**DOI:** 10.1016/j.vaccine.2016.03.039

**Published:** 2016-12-01

**Authors:** Robbie Kerr, Linda O. Eckert, Beverly Winikoff, Jill Durocher, Shireen Meher, Sue Fawcus, Shuchita Mundle, Ben Mol, Sabaratnam Arulkumaran, Khalid Khan, Julius Wandwabwa, Sonali Kochhar, Andrew Weeks, The Brighton Collaboration Primary Postpartum Haemorrhage Working Group

**Affiliations:** a University of Liverpool, UK; b University of Washington, WA, USA; c Gynuity Health Projects, NY, USA; d Mowbray Maternity Hospital, South Africa; e Government Medical College and Hospital Nagpur, India; f University of Adelaide, Australia; g St George's University of London, UK; h Barts and The London School of Medicine and Dentistry, UK; i Busitema University, Mbale Regional Referral Hospital, Uganda; j Global Healthcare Consulting, India

**Keywords:** Postpartum haemorrhage, Adverse event, Immunization, Guidelines, Case definition

## 1 Preamble

### 1.1 Need for developing case definitions and guidelines for data collection, analysis, and presentation for primary postpartum haemorrhage as an adverse event following immunization

Postpartum haemorrhage (PPH) describes excessive bleeding after delivery of a foetus. It is the leading cause of maternal death, responsible for approximately 68,500 deaths a year, 99.7% occurring in developing regions [1]. It occurs in approximately 6% of deliveries when defined as a blood loss equal to, or greater than 500 ml, or 1–2% when a 1000 ml is used [2]. It therefore represents a significant global health burden, disproportionately affecting those in the world's poorest countries.

There is increasing interest into a broader role for vaccination in the prevention of neonatal and pregnancy-related infections. However, it is vital that all vaccines are monitored for their potential adverse effects, including their effect on key obstetric complications, such as postpartum haemorrhage. There is a need therefore for consensus on a unifying definition of postpartum haemorrhage to be used in vaccine trials, epidemiological and safety studies. Our case definition aims to standardize the definition for research and adverse event reporting. The purpose of this definition is not to establish new clinical indicators or thresholds for deciding when to treat post partum haemorrhage.

Postpartum haemorrhage is the consequence of several different pathologies that can occur in isolation or combination: uterine atony, genital tract trauma, retained placental tissue and coagulation dysfunction. In severe cases of postpartum haemorrhage often pathologies co-exist, with intractable haemorrhage often leading to coagulopathy. Uterine atony is regarded as the most common cause of PPH. It occurs when inadequate myometrial tone results in unchecked blood flow to the placental bed.

An individual's risk of excessive blood loss will be influenced by numerous pre-existing, pregnancy-related and obstetric factors. Risk factors for PPH include: Asian ethnicity; obesity; previous PPH; multiple pregnancy; anaemia; large baby; placenta praevia; age over 40 years; induction of labour; prolonged labour; intrapartum pyrexia; placental abruption; episiotomy; operative vaginal delivery; retained placenta; and delivery by caesarean section [3]. Incidence and severity of PPH will therefore vary widely depending on the population studied and the obstetric practice. PPH-related adverse clinical events will also vary depending on the individual. For example the same blood loss could have no clinical consequence in a healthy woman, but be a life-threatening event for in a woman with severe anaemia.

The accurate quantification of blood loss can be challenging. Estimation of blood loss has been shown to be inaccurate, with underestimation worsening at larger volumes [4], [5]. Photometric methods, where the haemoglobin content of all swabs, drapes and pads at delivery is examined in laboratory, offer the greatest accuracy [6]. The cost and logistics of these methods however mean they are used only in niche research studies. Direct measurement with an under-buttock, calibrated drape correlates with lab-based methods; although its accuracy depends on timely placement to collect blood without amniotic fluid or urine [7]. Unfortunately the routine use of a calibrated drape is not associated with a decrease in severe PPH, therefore their routine use is mainly limited to research [8].

A World Health Organization technical working group in 1989 endorsed a definition of a blood loss of “500 ml or more from the genital tract after delivery of the baby” [9]. They accepted that this was “an arbitrary figure” and “not always of great clinical significance;” however, they decided against a greater volume as “the measurement of collected blood frequently and significantly underestimates the actual blood loss.”

The Royal College of Obstetricians and Gynaecologists (UK) also endorse a definition based on 500 ml or more [3]. However, in the absence of shock, they recommend only “readiness for resuscitation” at this blood loss. If “major PPH” occurs, defined by a blood loss 1000 ml or greater, they recommend “a full protocol of measures to achieve resuscitation and haemostasis.” Similarly, the American Congress of Obstetricians and Gynaecologists endorses a definition of 1000 ml or more “or any loss with symptoms or signs of hypovolemia” [10].

Randomized trials investigating PPH treatments have used a range of blood loss volumes in their inclusion criteria, to identify women that could benefit from treatment: 500 ml [11], [12], [13], [14], 700 ml [15], [16], 800 ml [17], 1000 ml [18] and 1500 ml [19]. Other authors have used a fall in haemoglobin or haematocrit as a definition, to avoid the difficulties of measurement or estimation of blood loss [20], [21]. The Cochrane Collaboration used blood loss of 1000 ml or more and maternal mortality as their primary outcomes in their reviews of PPH preventions [22], [23]. Non-Cochrane reviews and randomized trials of PPH preventions have used treatment-based criteria, such as the requirement of transfusion or additional uterotonics [24].

There is hence no uniformly accepted definition of postpartum haemorrhage to use in a vaccination trials and surveillance systems. This is a missed opportunity as a definition would enhance data comparability, facilitate data interpretation and promote the scientific understanding of postpartum haemorrhage as an adverse event.

### 1.2 Methods for the development of the case definition and guidelines for data collection, analysis, and presentation for postpartum haemorrhage as an adverse events following immunization

Following the process described on the Brighton Collaboration Website http://www.brightoncollaboration.org/internet/en/index/process.html, the Brighton Collaboration *Postpartum Haemorrhage Working Group* was formed in 2015 and included members of clinical and academic, but also public health and epidemiology background. The composition of the working and reference group as well as results of the web-based survey completed by the reference group with subsequent discussions in the working group can be viewed at: http://www.brightoncollaboration.org/internet/en/index/working_groups.html.

To guide the decision-making for the case definition and guidelines, a literature search was performed using the Cochrane library, Medline (via Ovid), the Web of Knowledge and Scopus, including the terms [postpartum haemorrhage OR postpartum haemorrhage] AND defini-. The search resulted in the identification of 295 references. A further 24 references were found by searching of reference lists, by web-search and through recommendation by working group members. All abstracts were screened for definitions for postpartum haemorrhage where a reference or rationale was given. All abstracts were in English language. 47 articles with potentially relevant material were reviewed in full, in order to identify studies using case definitions. This review resulted in a detailed summary of 42 articles, including information on the study design, the definition put forth, the rationale or references given, and any alternative definitions mentioned. In addition, a summary of primary outcomes used in trials evaluating interventions to prevent PPH; and a summary of diagnostic criteria used in PPH treatment trials, were distributed to working group members.

An inventory comprising of 34 relevant definitions of postpartum haemorrhage was made available to working group members.

### 1.3 Rationale for selected decisions about the case definition of postpartum haemorrhage as an adverse event following immunization

*The term “postpartum haemorrhage”*

Postpartum haemorrhage is defined by ICD-10 as “haemorrhage after delivery of a foetus or infant” (ICD-10). If some bleeding after delivery is universally expected, the key question is when does bleeding become a haemorrhage, and normality become disease?

“Postpartum haemorrhage” is a widely accepted term, despite lack of consensus on its definition. Authors and professional societies often use adjunctive terms (e.g. severe, major, massive) to denote increased severity, but the use of these terms can also be inconsistent [3], [25], [26], [27], [28], [29]. There appears to be almost universal acceptance of the terms “early” or “primary” to describe postpartum haemorrhage that occurs within 24 h of delivery, and “secondary” to describe haemorrhage occurring thereafter up to 42 days post-delivery. For the purposes of monitoring adverse events following vaccination, both primary and secondary postpartum haemorrhages are of potential interest. The working group therefore decided on the case definition of “postpartum haemorrhage” as a term in isolation.

It is entirely appropriate for postpartum haemorrhage to have different definitions depending on the purpose for which it is to be used. If used as a threshold for a birth attendant calling for additional help, then a low volume of blood loss should be used, to facilitate early treatment of the cause. If used for clinical trials, audit or research then definitions using higher volumes of blood loss and markers of maternal morbidity are of greater interest. The definition in this document is for the latter purpose, and must not be mistaken as a clinical guide for when to start treatment.

Postpartum blood loss only becomes of interest to the individual when it causes symptoms or some dysfunction in the new mother. We therefore have interest if it causes symptoms or affects the function as a continuum from “normal” to “haemorrhage”. There is not a natural threshold.

To the new mother, postpartum blood loss is only significant if it causes symptoms or an adverse clinical event. We therefore have focused this case definition on genital tract bleeding that “leads to an adverse clinical outcome.” In the definition presented below, Level I, with the highest specificity also represents the highest severity. Within the definition context, however, the three diagnostic levels must not be simply misunderstood as reflecting different grades of clinical severity. They instead reflect diagnostic certainty (see below). The availability of diagnostic tools or treatments will vary depending on the site. This may determine what level of diagnostic certainty can be reached, although all levels are considered acceptable.  *Adverse clinical outcomes*

In clinical practice it is not the precise volume of blood loss that is of concern, but rather the clinical condition of the woman and the response of her blood loss to treatment. Postpartum bleeding may result in symptoms of hypovolemia or anaemia e.g. exertional dyspnoea, postural presyncope, tiredness or reduced consciousness. At the furthest extreme uncorrected hypovolaemic shock can lead to organ-dysfunction and maternal death.

Fortunately, in most settings, the initiation of resuscitation and haemostatic measures prevents PPH morbidity and mortality. Management is often given in steps, assessing response to less invasive, lower risk treatments first, before moving onto invasive surgical interventions. As a result, use of these latter interventions for postpartum haemorrhage can be used as a highly specific criterion for a case definition.

The Working Group decided that genital tract bleeding that leads to a maternal death, or maternal near miss (as defined by WHO [30]) warrants Level I, the highest certainty for postpartum haemorrhage.  *Blood loss volumes*

The use of blood loss volume is the long-established method to define PPH [3], [9], [10]. If the blood loss is known then a volume cut-off can be used to define when a PPH has occurred. The challenge is in determining where that cut-off should lie.

The working group hypothesized that there could be a single blood loss volume cut-off that represents the optimum sensitivity and specificity for an adverse clinical outcome in a given population e.g. blood transfusion for PPH. To our knowledge there have been no published studies presenting this analysis, or describing the incidence of adverse events at each volume of blood loss. However, unpublished data from a trial that routinely measured blood loss at 33, 055 deliveries, revealed the six patients with serious adverse events (hysterectomy or death) all had blood losses greater than 1000 ml [16].

The reverse approach is to examine the “normal” blood loss in a low-risk population; and define PPH as an upper centile of this. This requires deliveries without third-stage prophylaxis, as it is proven to decrease mean blood loss but is not available in every setting. The working group was given access to unpublished data of measured blood losses from 9348 low-risk vaginal deliveries with no prophylaxis and no treatment until blood loss exceeded 700 ml [15]. Blood losses of at least 500 ml, 700 ml and 1000 ml represented the 84th, 89th and 97th centiles respectively.

The working group agreed that it is fair to assume the likelihood of adverse clinical outcomes increases with higher blood losses. As no studies have validated the use of a volume cut-offs and any choice of a centile is arbitrary, it was agreed to endorse an already established cut-off. This definition uses 1000 ml or more. It is specific as it is a relatively rare occurrence, and sensitive as significant adverse clinical outcomes are unlikely under this level.  *Blood loss measurement or estimation*

In response to the well-known inaccuracies of estimation, this definition gives a greater level of diagnostic certainty to measured blood loss of 1000 ml or more (level 2). Estimated blood loss of 1000 ml or more is included at level 3, as there are many settings where measurement is not possible.

The working group recommends direct measurement of blood loss by using a calibrated plastic drape placed under the buttocks immediately following delivery over other methods [31]. Gravimetric, or decanting a bed-pan into a measuring jug, may also be used if care is taken to collect all the blood without contamination. Weighing must be undertaken immediately, or the delivery swabs placed in a sealed container, to avoid evaporation.  *Clinical signs – vital signs*

There will be instances where blood loss is difficult to assess, not documented, or not known (e.g. with patient transferred from the community to a facility). The working group agreed that these cases should fit into definition, especially when they experience adverse outcomes or their vital signs indicate hypovolemic shock.

In postpartum haemorrhage there is limited evidence validating vital sign cut-offs. A recent systematic review on the relationship between blood loss and clinical signs was unable to establish specific vital sign cut-offs for interventions in PPH [32]. Shock index (heart rate divided by systolic blood pressure) has been shown to be a better predictor of adverse outcome in postpartum haemorrhage, than other vital signs [33], [34]; but few studies have validated a cut-off to be used [27].

The working group acknowledged the promise of shock index, although agreed the present evidence was insufficient for inclusion. The definition uses hypotension with genital tract bleeding at Level 2, as this has high specificity, and is already endorsed by WHO in their definition of severe postpartum haemorrhage (WHO).  *Influence of treatment on fulfilment of case definition*

The case definition uses treatment at levels 1 and 2. Genital bleeding that leads to a hysterectomy or transfusion of ≥5 units of blood reaches level 1 of certainty in this definition [30]. These life-saving interventions use significant resources and are associated with their own morbidity; therefore it can be assumed their use represents a high specificity for postpartum haemorrhage.

The requirement of blood transfusion is a valuable proxy for clinical concern, especially as its availability is limited in many settings. Practitioners may advise transfusion in response to clinical signs, perceived volume or rate of blood loss, laboratory investigations, or patient symptoms. The consensus was for any blood transfused in response to genital bleeding to meet level two certainty of the case definition.

First-line interventions in postpartum haemorrhage, for example uterotonic medications or intravenous fluids, are not used in this definition. These treatments may be initiated in response to a perceived abnormal fast rate of blood loss that arrests well before the 1000 ml threshold. The use and availability of these interventions will vary greatly between different settings.  *Laboratory examinations – haemoglobin and haematocrit*

The working group decided that a fall in haemoglobin or haematocrit measured before and after delivery did not have a place in this definition. Although it represents an objective alternative to estimated blood loss, it will be altered by the patient's fluid status at the time of testing. There exists no consensus for the timing of the postnatal blood test and the investigator will face the logistical challenge of blood collection a fixed number of hours following delivery. In addition, this approach is not valid when patients require a blood transfusion acutely for postpartum haemorrhage. Finally, laboratory testing is not available in many settings.  *Formulating a case definition that reflects diagnostic certainty: weighing specificity versus sensitivity*

The case definition has been formulated such that the Level 1 definition is highly specific for the condition. As maximum specificity normally implies a loss of sensitivity, two additional diagnostic levels have been included in the definition, offering a stepwise increase of sensitivity from Level 1 down to Level 3, while retaining an acceptable level of estimated specificity at all levels. In this way it is hoped that all possible cases of postpartum haemorrhage can be captured.

It needs to be re-emphasized that the grading of definition levels is entirely about diagnostic certainty, not clinical severity of an event. Thus, a clinically very severe event may appropriately be classified as Level 2 or 3 rather than Level One. Information about the severity of the event should additionally always be recorded, as specified by the data collection guidelines.  *Timing post immunization*

A specific time frame for postpartum haemorrhage following immunization is not included as the literature review revealed no case reports or studies where a relationship was suggested. We postulate that a definition designed to be a suitable tool for testing causal relationships requires ascertainment of the outcome (e.g. postpartum haemorrhage) independent from the exposure (e.g. immunizations). Therefore, to avoid selection bias, a restrictive time interval from immunization to onset of postpartum haemorrhage should not be an integral part of such a definition. Instead, where feasible, details of this interval should be assessed and reported as described in the data collection guidelines.

Further, postpartum haemorrhage often occurs outside the controlled setting of a clinical trial or hospital. In some settings it may be impossible to obtain a clear timeline of the event, particularly in less developed or rural settings. In order to avoid selecting against such cases, the Brighton Collaboration case definition avoids setting arbitrary time frames.  **Guidelines for data collection, analysis and presentation**

As mentioned in the overview paper, the case definition is accompanied by guidelines which are structured according to the steps of conducting a clinical trial, i.e. data collection, analysis and presentation. Neither case definition nor guidelines are intended to guide or establish criteria for management of ill infants, children, or adults. Both were developed to improve data comparability.

### 1.4 Periodic review

Similar to all Brighton Collaboration case definitions and guidelines, review of the definition with its guidelines is planned on a regular basis (i.e. every three to five years) or more often if needed.

## 2 Case definition of postpartum haemorrhage

Postpartum haemorrhage is a condition characterized by

**Level 1 of diagnostic certainty**

• Genital bleeding after delivery leading to severe maternal outcome (maternal death or maternal near miss) as defined by WHO [28].

**Level 2 of diagnostic certainty**

• Genital bleeding after delivery with at least one of the following: measured abnormal bleeding (1000 ml or more), or any bleeding leading to hypotension or blood transfusion.

**Level 3 of diagnostic certainty**

• Genital bleeding after delivery estimated at 1000 ml or more

**Terminology**

“Delivery” is the birth of an offspring, which breathes or shows evidence of life, or is born without signs of life at an estimated gestation of 24 weeks or more (as defined by *Preterm Birth* guidelines)(http://www.brightoncollaboration.org).

“Maternal death” is the death of a woman while pregnant or within 42 days of termination of pregnancy, irrespective of the duration and the site of the pregnancy (as defined by *Maternal Death* guidelines (http://www.brightoncollaboration.org)).

## 3 Guidelines for data collection, analysis and presentation of postpartum haemorrhage

It was the consensus of the Brighton Collaboration *Postpartum Haemorrhage Working Group* to recommend the following guidelines to enable meaningful and standardized collection, analysis, and presentation of information about postpartum haemorrhage. However, implementation of all guidelines might not be possible in all settings. The availability of information may vary depending upon resources, geographical region, and whether the source of information is a prospective clinical trial, a post-marketing surveillance or epidemiological study, or an individual report of postpartum haemorrhage. Also, as explained in more detail in the overview paper in this volume, these guidelines have been developed by this working group for guidance only, and are not to be considered a mandatory requirement for data collection, analysis, or presentation.

### 3.1 Data collection

These guidelines represent a desirable standard for the collection of data on availability following immunization to allow for comparability of data, and are recommended as an addition to data collected for the specific study question and setting. The guidelines are not intended to guide the primary reporting of Postpartum haemorrhage to a surveillance system or study monitor. Investigators developing a data collection tool based on these data collection guidelines also need to refer to the criteria in the case definition, which are not repeated in these guidelines. The Brighton Collaboration has developed guidelines for data collection https://brightoncollaboration.org/public/resources/standards/guidelines.html; and data collection forms https://brightoncollaboration.org/public/resources/data-collection-forms.html.

Guidelines numbers below have been developed to address data elements for the collection of adverse event information as specified in general drug safety guidelines by the International Conference on Harmonization of Technical Requirements for Registration of Pharmaceuticals for Human Use [35], and the form for reporting of drug adverse events by the Council for International Organizations of Medical Sciences [36]. These data elements include an identifiable reporter and patient, one or more prior immunizations, and a detailed description of the adverse event, in this case, of postpartum haemorrhage following immunization. The additional guidelines have been developed as guidance for the collection of additional information to allow for a more comprehensive understanding of postpartum haemorrhage following immunization.

#### 3.1.1 Source of information/reporter

For all cases and/or all study participants, as appropriate, the following information should be recorded:

1) Date of report.
2) Name and contact information of person reporting^2^ and/or diagnosing the postpartum haemorrhage as specified by country-specific data protection law.
3) Name and contact information of the investigator responsible for the subject, as applicable.
4) Relation to the patient (e.g., immunizer [clinician, nurse], family member [indicate relationship], other).

#### 3.1.2 Vaccine/control

##### 3.1.2.1 Demographics

For all cases and/or all study participants, as appropriate, the following information should be recorded:

5) Case/study participant identifiers (e.g. first name initial followed by last name initial) or code (or in accordance with country-specific data protection laws).
6) Date of birth, age, sex, race and ethnicity.
7) For infants: Gestational age and birth weight.

##### 3.1.2.2 Clinical and immunization history

For all cases and/or all study participants, as appropriate, the following information should be recorded:

8) Past medical history, including hospitalizations, underlying diseases/disorders, pre-immunization signs and symptoms e.g. anaemia, fibroids, previous surgery, known or suspected coagulopathies including platelet disorders. Past obstetric history: parity, previous modes of delivery, previous PPH, previous retained placenta. Obstetric history: gestation; presence of antepartum bleeding; if induced- gestation and methods used; timing of membrane rupture- spontaneous or amniotomy; duration of first, second and third stages of labour; presence of intrapartum pyrexia; time and place of delivery; staff present at delivery; mode of delivery; use of episiotomy; if operative-staff cadre performing; birth weight; live or stillbirth; use of PPH prophylaxis; presence of retained tissue; staff present during treatment.
9) Any medication history (other than treatment for the event described) prior to, during, and after immunization including prescription and non-prescription medication as well as medication or treatment with long half-life or long term effect. (e.g. immunoglobulins, blood transfusion and immunosuppressants).
10) Immunization history (i.e. previous immunizations and any adverse event following immunization (AEFI)).

#### 3.1.3 Details of the immunization

For all cases and/or all study participants, as appropriate, the following information should be recorded:

11) Date and time of immunization(s).
12) Description of vaccine(s) (name of vaccine, diluent, manufacturer, lot number, dose (e.g. 0.25 mL, 0.5 mL, etc.) and number of dose if part of a series of immunizations against the same disease).
13) The anatomical sites (including left or right side) of all immunizations (e.g. vaccine A in proximal left lateral thigh, vaccine B in left deltoid).
14) Route and method of administration (e.g. intramuscular, intradermal, subcutaneous, and needle-free (including type and size), other injection devices).
15) Needle length and gauge.

#### 3.1.4 The adverse event

16) For all cases at any level of diagnostic certainty and for reported events with insufficient evidence, the criteria fulfilled to meet the case definition should be recorded.
  Specifically document:
17) Clinical description of signs and symptoms of postpartum haemorrhage, and if there was medical confirmation of the event (i.e. patient seen by physician).
18) Date/time of onset,^3^ first observation^4^ and diagnosis,^5^ end of episode^6^ and final outcome.^7^
19) Concurrent signs, symptoms, and diseases.
20) Measurement/testing
• The primary assessment is of postnatal volume of blood loss. We would recommend the calibrated under-buttock drape e.g. BRASSS-V for direct measurement of blood loss. In the absence of this, blood can be measured using a “fracture” bed-pan and measuring jug, or weighing of absorbent materials.
• Table 1 gives a list of optimal clinical, laboratory and management criteria to be assessed in order to diagnose a WHO maternal near-miss.
• Values and units of routinely measured parameters (e.g. blood pressure, heart rate, blood loss) – in particular those indicating the severity of the event. Hypotension in Level 2 of our case definition should be defined as a systolic blood pressure of under 90 mmHg.
• Method of measurement (e.g. type of sphygmometer, timing of measurement, patient position, for blood loss-calibrated under-buttock drape, bed pan and jug or gravimetric etc.);
• Results of laboratory examinations (especially haemoglobin, haematocrit, platelet count and coagulation screen), surgical and/or pathological findings and diagnoses if present.
21) Treatment given for postpartum haemorrhage, especially oxytocin; ergometrine; prostaglandins including misoprostol; bimanual compression; aortic compression; transfusion with whole blood; red cell concentrate; fresh frozen plasma; fibrinogen; clotting factors; and invasive surgical intervention (including manual removal of placenta).
22) Outcome^8^ at last observation.
23) Objective clinical evidence supporting classification of the event as “serious” 9.
24) Exposures other than the immunization 24 h before and after immunization (e.g. food, environmental) considered potentially relevant to the reported event.

#### 3.1.5 Miscellaneous/general

25) The duration of surveillance for postpartum haemorrhage should be predefined based on
26) Biologic characteristics of the vaccine e.g. live attenuated versus inactivated component vaccines;
27) Biologic characteristics of the vaccine-targeted disease;
28) Biologic characteristics of postpartum haemorrhage including patterns identified in previous trials (e.g. early-phase trials); and
29) Biologic characteristics of the vaccine (e.g. nutrition, underlying disease like immunodepressing illness).
30) The duration of follow-up reported during the surveillance period should be predefined likewise. It should aim to continue to resolution of the event.
31) Methods of data collection should be consistent within and between study groups, if applicable.
32) Follow-up of cases should attempt to verify and complete the information collected as outlined in data collection guidelines 1–24.
33) Investigators of patients with postpartum haemorrhage should provide guidance to reporters to optimize the quality and completeness of information provided.
34) Reports of postpartum haemorrhage should be collected throughout the study period regardless of the time elapsed between immunization and the adverse event. If this is not feasible due to the study design, the study periods during which safety data are being collected should be clearly defined.

### 3.2 Data analysis

The following guidelines represent a desirable standard for analysis of data on postpartum haemorrhage to allow for comparability of data, and are recommended as an addition to data analyzed for the specific study question and setting.

31) Reported events should be classified in one of the following five categories including the three levels of diagnostic certainty. Events that meet the case definition should be classified according to the levels of diagnostic certainty as specified in the case definition. Events that do not meet the case definition should be classified in the additional categories for analysis.

**Event classification in 5 categories**^10^

**Event meets case definition**

1) Level 1: Criteria as specified in the postpartum haemorrhage case definition
2) Level 2: Criteria as specified in the postpartum haemorrhage case definition
3) Level 3: Criteria as specified in the postpartum haemorrhage case definition

**Event does not meet case definition**

***Additional categories for analysis***

4) Reported postpartum haemorrhage (either related to blood loss volume or use of therapy) with insufficient evidence to meet the case definition^11^
5) Not a case of postpartum haemorrhage

32) The interval between immunization and reported postpartum haemorrhage could be defined as the date/time of immunization to the date/time of onset^3^ of the first symptoms and/or signs consistent with the definition. If few cases are reported, the concrete time course could be analyzed for each; for a large number of cases, data can be analyzed in the following increments:

**Subjects with PPH by Interval from immunization to PPH**

**Table:** 

**Interval between immunization and PPH**	**Number**
0–72 h after immunization	
More than 72 h to 7 days after immunization	
More than 7 days to 30 days after immunization	
More than 30 days after immunization (pregnant at time)	
Vaccination prior to pregnancy	
**Total**

33) The duration of a possible postpartum haemorrhage could be analyzed as the interval between the date/time of onset^2^ of the first symptoms and/or signs consistent with the definition and the end of episode^6^ and/or final outcome^7^. Whatever start and ending are used, they should be used consistently within and across study groups.
34) If more than one measurement of a particular criterion is taken and recorded, the value corresponding to the greatest magnitude of the adverse experience could be used as the basis for analysis. Analysis may also include other characteristics like qualitative patterns of criteria defining the event.
35) The distribution of data (as numerator and denominator data) could be analyzed in predefined increments (e.g. measured values, times), where applicable. Increments specified above should be used. When only a small number of cases are presented, the respective values or time course can be presented individually.
36) Data on postpartum haemorrhage obtained from subjects receiving a vaccine should be compared with those obtained from an appropriately selected and documented control group(s) to assess background rates of hypersensitivity in non-exposed populations, and should be analyzed by study arm and dose where possible, e.g. in prospective clinical trials.

### 3.3 Data presentation

These guidelines represent a desirable standard for the presentation and publication of data on postpartum haemorrhage following immunization to allow for comparability of data, and are recommended as an addition to data presented for the specific study question and setting. Additionally, it is recommended to refer to existing general guidelines for the presentation and publication of randomized controlled trials, systematic reviews, and meta-analyses of observational studies in epidemiology (e.g. statements of Consolidated Standards of Reporting Trials (CONSORT), of Improving the quality of reports of meta-analyses of randomized controlled trials (QUORUM), and of Meta-analysis Of Observational Studies in Epidemiology (MOOSE), respectively) [37], [38], [39].

37) All reported events of postpartum haemorrhage should be presented according to the categories listed in guidelines.
38) Data on possible postpartum haemorrhage events should be presented in accordance with data collection guidelines 1–24 and data analysis guidelines 31–36.
39) Terms to describe postpartum haemorrhage such as “low-grade”, “mild”, “moderate”, “high”, “severe” or “significant” are highly subjective, prone to wide interpretation, and should be avoided, unless clearly defined.
40) Data should be presented with numerator and denominator (*n*/*N*) (and not only in percentages), if available.

Although immunization safety surveillance systems denominator data are usually not readily available, attempts should be made to identify approximate denominators. The source of the denominator data should be reported and calculations of estimates be described (e.g. manufacturer data like total doses distributed, reporting through Ministry of Health, coverage/population based data, etc.).

41) The incidence of cases in the study population should be presented and clearly identified as such in the text.
42) If the distribution of data is skewed, median and range are usually the more appropriate statistical descriptors than a mean. However, the mean and standard deviation should also be provided.
43) Any publication of data on postpartum haemorrhage should include a detailed description of the methods used for data collection and analysis as possible. It is essential to specify:
• The study design;
• The method, frequency and duration of monitoring for postpartum haemorrhage
• The trial profile, indicating participant flow during a study including drop-outs and withdrawals to indicate the size and nature of the respective groups under investigation;
• The type of surveillance (e.g. passive or active surveillance);
• The characteristics of the surveillance system (e.g. population served, mode of report solicitation);
• The search strategy in surveillance databases;
• Comparison group(s), if used for analysis;
• The instrument of data collection (e.g. standardized questionnaire, report form);
• Whether the day of immunization was considered “day one” or “day zero” in the analysis;
• Whether the date of onset^3^ and/or the date of first observation^4^ and/or the date of diagnosis^5^ was used for analysis; and
• Use of this case definition for postpartum haemorrhage, in the abstract or methods section of a publication.^12^

## Figures and Tables

**Table 1:** The WHO maternal near miss criteria [28].

**Clinical criteria**	Acute cyanosis Gasping^a^ Respiratory rate >40 or <6 min^−1^ **Shock**^b^ **Oliguria non responsive to fluids or diuretics**^c^ **Clotting failure**^d^ Loss of consciousness lasting ≥12 h^e^ Loss of consciousness AND absence of pulse/heart beat Stroke^f^ Uncontrollable fit/total paralysis^g^ Jaundice in the presence of pre-eclampsia^h^
**Laboratory-based criteria**	Oxygen saturation <90% for ≥60 min pH < 7.1 PaO_2_/FiO_2_ <200 mmHg Lactate >5 Creatinine >300 mmol/l or >3.5 mg/dl Acute thrombocytopenia (<50,000 platelets) Bilirubin >100 mmol/l or >6.0 mg/dl Loss of consciousness AND the presence of glucose and ketoacids in urine
**Management-based criteria**	Use of continuous vasoactive drugs^i^ Intubation and ventilation for ≥60 min not related to anaesthesia **Hysterectomy following** infection or **haemorrhage** Dialysis for acute renal failure **Transfusion of ≥5 units red cell transfusion** Cardio-pulmonary resuscitation (CPR)

A woman presenting with any of the following life-threatening conditions and surviving a complication that occurred during pregnancy, childbirth or within 42 days of termination of pregnancy should be considered as a maternal near miss case. (Criterion most relevant to postpartum haemorrhage in bold).

a Gasping is a terminal respiratory pattern and the breath is convulsively and audibly caught.

b Shock is a persistent severe hypotension, defined as a systolic blood pressure <90 mmHg for ≥60 min with a pulse rate at least 120 despite aggressive fluid replacement (>2l).

c Oliguria is defined as an urinary output <30 ml/h for 4 h or <400 ml/24 h.

d Clotting failure can be assessed by the bedside clotting test or absence of clotting from the IV site after 7–10 min.

e Loss of consciousness is a profound alteration of mental state that involves complete or near-complete lack of responsiveness to external stimuli. It is defined as a Coma Glasgow Scale <10 (moderate or severe coma).

f Stroke is a neurological deficit of cerebrovascular cause that persists beyond 24 h or is interrupted by death within 24 h.

g Condition in which the brain is in a state of continuous seizure.

h Pre-eclampsia is defined as the presence of hypertension associated with proteinuria. Hypertension is defined as a blood pressure of at least 140 mmHg (systolic) or at least 90 mmHg (diastolic) on at least two occasions and at least 4–6 h apart after the 20th week of gestation in women known to be normotensive beforehand. Proteinuria is defined as excretion of 300 mg or more of protein every 24 h. If 24-h urine samples are not available, proteinuria is defined as a protein concentration of 300 mg/l or more (≥1+ on dipstick) in at least two random urine samples taken at least 4–6 h apart.

i For instance, continuous use of any dose of dopamine, epinephrine or norepinephrine.

## Footnotes

2 If the reporting centre is different from the vaccinating centre, appropriate and timely communication of the adverse event should occur.

3 The date and/or time of onset are defined as the time post immunization, when the first sign or symptom indicative for postpartum haemorrhage occurred. This may only be possible to determine in retrospect.

4 The date and/or time of first observation of the first sign or symptom indicative for postpartum haemorrhage can be used if date/time of onset is not known.

5 The date of diagnosis of an episode is the day post immunization when the event met the case definition at any level.

6 The end of an episode is defined as the time the event no longer meets the case definition at the lowest level of the definition.

7 E.g. recovery to pre-immunization health status, spontaneous resolution, therapeutic intervention, persistence of the event, sequelae, death.

8 An AEFI is defined as serious by international standards if it meets one or more of the following criteria: (1) it results in death, (2) is life-threatening, (3) it requires inpatient hospitalization or results in prolongation of existing hospitalization, (4) results in persistent or significant disability/incapacity, (5) is a congenital anomaly/birth defect, (6) is a medically important event or reaction.

9 To determine the appropriate category, the user should first establish, whether a reported event meets the criteria for the lowest applicable level of diagnostic certainty, e.g. Level three. If the lowest applicable level of diagnostic certainty of the definition is met, and there is evidence that the criteria of the next higher level of diagnostic certainty are met, the event should be classified in the next category. This approach should be continued until the highest level of diagnostic certainty for a given event could be determined. Major criteria can be used to satisfy the requirement of minor criteria. If the lowest level of the case definition is not met, it should be ruled out that any of the higher levels of diagnostic certainty are met and the event should be classified in additional categories four or five.

10 If the evidence available for an event is insufficient because information is missing, such an event should be categorized as “Reported postpartum haemorrhage with insufficient evidence to meet the case definition”.

11 An event does not meet the case definition if investigation reveals a negative finding of a necessary criterion (necessary condition) for diagnosis. Such an event should be rejected and classified as “Not a case of postpartum haemorrhage”.

12 Use of this document should preferably be referenced by referring to the respective link on the Brighton Collaboration website (http://www.brightoncollaboration.org).
